# Efficacy of Faradic Foot Baths and Short Foot Exercises in Symptomatic Flatfoot: A Review

**DOI:** 10.7759/cureus.47803

**Published:** 2023-10-27

**Authors:** Anushri R Patil, Swapna Jawade, Kamya J Somaiya, Manali A Boob

**Affiliations:** 1 Physiotherapy, Ravi Nair Physiotherapy College, Datta Meghe Institute of Higher Education and Research, Wardha, IND

**Keywords:** rehabilitation, medial longitudinal arch, faradic foot bath, short foot exercises, flat foot

## Abstract

Flatfoot is a common condition among adults, according to orthopedic experts. Flatfoot is defined in this document as a foot condition that occurs after the completion of skeletal growth and is characterized by either partial or complete reduction of the medial longitudinal arch (MLA). The purpose of this study was to evaluate the effects of short foot exercise (SFE) and faradic foot baths on people who have flat feet. This review focused on comprehensive original primary articles written in English. Numerous studies have been conducted in order to determine the effects of both interventions. The search yielded a wide range of papers, including editorials, review articles, freely available full texts, and abstracts. The results showed that both SFE and faradic foot baths effectively improved flat feet.

## Introduction and background

According to orthopedics, flatfoot is a common condition in adults. Adult flatfoot is defined in this review as a foot condition that develops or persists after the bones have matured, characterized by a total or partial weakening of the medial longitudinal arch (MLA). Adult flatfoot can appear as an unexpected discovery or a state with symptoms, resulting in a range of clinical effects ranging from minor restrictions to significant disability and significant pain that significantly interferes with daily life activities [1,2]. Within the active subsystem, the extrinsic and intrinsic muscles of the foot work together to uphold the MLA. Intrinsic foot muscles with attachments spanning from the foot's origin to insertion points, like the abductor hallucis, flexor digitorum brevis, and quadratus plantae, assume essential responsibilities in maintaining the stability of the arch [3,4].

Pathological changes and symptoms arise due to alterations in the way structural loads are distributed across the medial foot and plantar arch, coupled with midfoot collapse and compression along the lateral column and rearfoot. Posterior tibial tendon insufficiency and peritalar subluxation refer to the abnormal misalignment of the talus around the subtalar and midtarsal joints and are the most common causes [5,6]. Assessing flatfoot needs a relevant patient history encompassing the emergence of the deformity and the duration and intensity of symptoms, with particular attention to pain in the arch and rearfoot. Additionally, it's feasible to uncover a familial background of flatfoot deformity [6,7]. In adults with flatfoot, concurrent conditions like rheumatoid arthritis, seronegative arthropathies, hypertension, or diabetes could hold significance. Elements such as occupation, level of activity, and obesity might also play a role. It's important to take into account factors like footwear, history of injuries, and prior treatments. A comprehensive assessment of various systems is essential, as this can impact everyday activities [8,9].

Associated symptoms like pain related to the hip, back, and knee are also linked to coinciding flatfoot conditions. In flexible adult flatfoot, the dropping of the longitudinal arch is associated with unusual inward rolling of the rearfoot when weight is borne. The talus moves inward and downward on the calcaneus, which tilts outward and downward simultaneously [10,11]. Pronation in the subtalar joint leads to an unstable midtarsal joint, contributing to different levels of sideways movement in the transverse plane [12,13]. Additional joints, like the tarsometatarsal joint, might also encounter an impact. Factors leading to pronation encompass adjusted inward angling of the forefoot (forefoot varus), flexible outward angling of the forefoot (forefoot valgus), restricted ankle movement (equinus), naturally occurring outward and upward angling of the heel and forefoot (congenital talipes calcaneovalgus), abnormal twisting involving inward or outward movement (adduction or abduction), muscle imbalance, laxity in ligaments, nerve-related foot conditions, and anything contributing to a shift of weight-bearing toward the inner side such as knock-knees, obesity, or wide gait [8,14]. The short foot exercise (SFE) is a therapeutic practice designed to enhance the strength of the intrinsic muscles in the foot. This technique involves pulling the head of the first metatarsal bone toward the heel, all while maintaining the toes in a neutral position without excessive bending or stretching.

It is suggested that incorporating SFE could enhance balance in functional movements among individuals with both normal and flat feet. This, in turn, may aid in preventing the descent of the navicular bone navicular drop by activating the intrinsic muscles [15,16]. An additional therapeutic approach for managing symptomatic flexible flat feet is the utilization of a faradic foot bath. This intervention alleviates the discomfort individuals encounter during their everyday tasks. It focuses on reinforcing the underdeveloped abductor muscles within the foot, which play a crucial role in upholding the foot's arch structure. This method employs a faradic current delivered through an electrode, offering adjustable levels of intensity and frequency. Furthermore, it enhances the strength of the feeble foot muscles [17].

Pathophysiology

Children commonly experience developmental flatfoot. Developmental flatfoot can have various origins, whether symptomatic or asymptomatic, characterized by flexibility or rigidity. Abnormal bone and joint development can lead to conditions like tarsal coalition or congenital vertical talus. Additionally, generalized ligament hyperflexibility from conditions such as Marfan or Ehlers-Danlos can contribute to flatfoot malformation. In adults, flatfoot deformity is categorized as either developmental or acquired. Acquired flatfoot is linked to tightness in the triceps surae or isolated gastrocnemius, traumatic deformities, dysfunction of the posterior tibial tendon, Charcot foot, midfoot laxity, external rotation of the hindfoot, ruptured plantar fascia, talus subluxation, or neuromuscular imbalances (such as polio, cerebral palsy). The multitude of factors contributing to this deformity can make pinpointing the precise cause of flatfoot in each scenario challenging [14,18,19].

Clinical manifestation

Adult flatfoot can arise from various pathological causes, including benign processes that result from ongoing congenital issues, traumatic events, or conditions linked to systemic health. The underlying pathology and associated symptoms stem from structural changes in how the plantar arch and medial foot bear loads, leading to collapse in the midfoot and constriction along the rearfoot and lateral column. Excessive use can lead to fatigue and cramping in the leg and foot muscles, contributing to the depression of the MLA, heel eversion or valgus positioning during stance in a relaxing manner, and forefoot abduction concerning the rearfoot. Notable areas of tenderness can be pinpointed through careful examination and palpation of the leg, ankle, and foot. These clinical traits frequently characterize individuals with flatfoot [3,7].

Diagnostic investigation

The primary criteria rely on the patient's history and clinical physical assessment, individuals who experience pain while weight bearing, walking, and walking in uncomfortable footwear. Diagnostic investigations are rarely required as flat feet can also be diagnosed on observation and physical assessment. Only patients with associated ankle and foot pathologies are advised to undergo examinations like MRI.

## Review

Study selection

**Figure 1:**
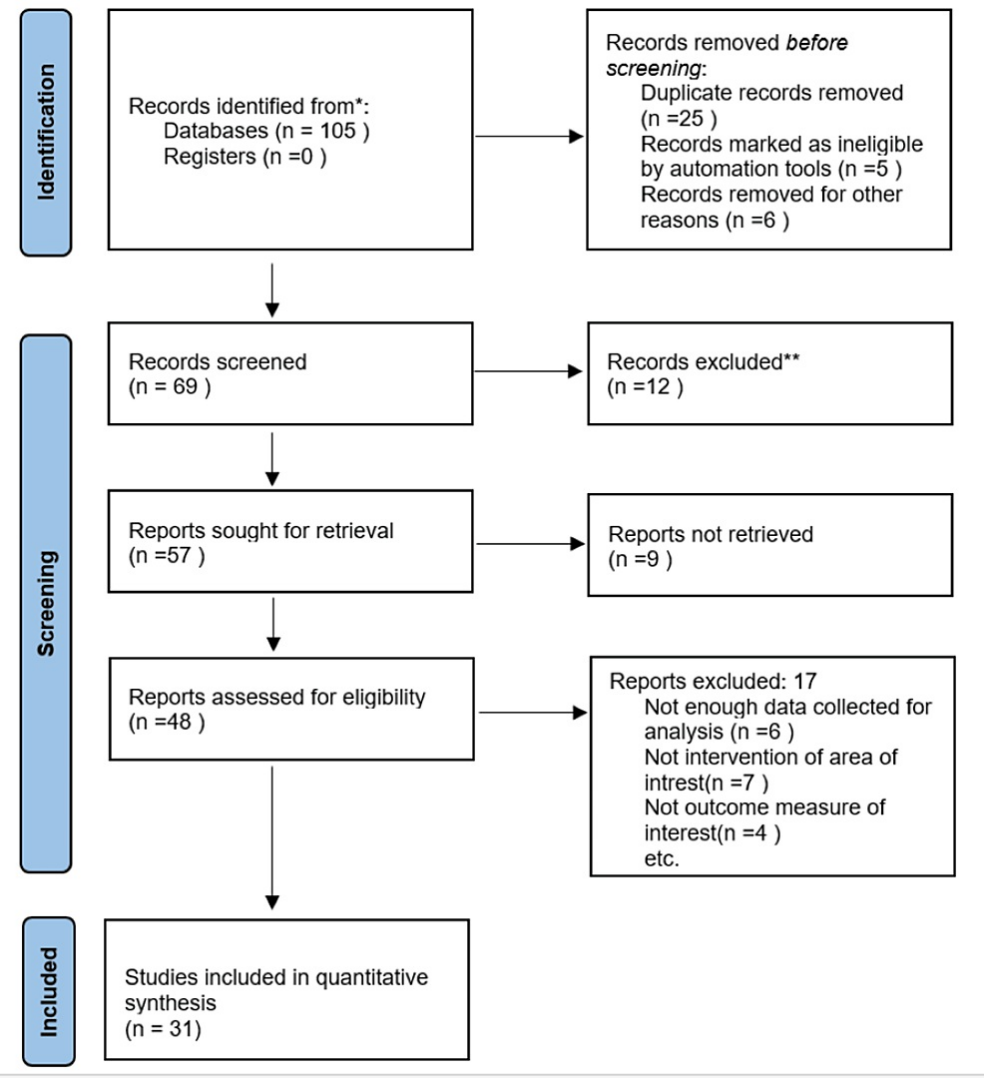
PRISMA flowchart PRISMA: Preferred Reporting Items for Systematic Reviews and Meta-Analysis

The search was performed using scholarly databases like PubMed, Google Scholar, and Science Direct using the keywords flatfoot, rehabilitation, faradic foot bath, and SFE. This review sought English-language, full-text primary original articles. Numerous papers, including editorials, review articles, free full texts, and abstracts, were found through the search. Data were extracted under the headings: author(year), sample size, population, outcome measures, and results were identified. The literature was then screened for relevant titles and abstracts. All the literature showed improvement in the flatfoot following the application of either a faradic foot bath or SFE. Out of the 105 articles, 31 were eligible for full-text review. The result of selecting articles is illustrated in Figure 1, which shows a search of the database and data extraction. Table 1 presents a summary of the articles that were reviewed for flatfoot.

**Table 1:** A summary of the articles reviewed for SFE and faradic foot bath used in flatfoot RCT: randomized control trial, ND: navicular drop, SFE: short foot exercise, AI: accuracy index, FPI: foot posture index, NH: navicular height, CSA: cross-sectional area, Abdh: abductor hallucis, NMES: neuromuscular electrical stimulation, ANC: antenatal care, mm: millimeter, SMT: sensorimotor training, LE: lower extremity, SFLE: short foot exercise combined with lower extremity training program, CPEI: center of the pressure excursion index, MLA: medial longitudinal arch

Author and year of publication	Study type (sample size)	Outcome measure	Intervention	Results	Conclusions
Huang et al. (2022) [3]	Meta-analysis of RCTs	ND, posture, muscle hypertrophy	Reviewed studies, RCT	There was a decrease in ND and muscle hypertrophy	It is recommended to use SFE for foot alignment in populations with flat feet
Kim and Lee (2020) [15]	A prospective non-randomized controlled trial (with 45 participants with flexible flat feet)	ND, static balance, AI of knee joint motion	SFE for five weeks	The static balance and perfection of knee joint motion were remarkably different pre- and post-exercise in the flatfoot group (p<0 .05)	SFE is found to be effective, along with visual feedback in improving flat feet
D'silva et al. (2017) [17]	An observational study (51 subjects with unilateral or bilateral flatfoot)	Staheli's arch index, FPI, NH	In Group A, talonavicular mobilization was administered. In Group B, low dye tapping was given. In Group C, a faradic foot bath was provided over a five-day period within a span of three weeks. All three groups also included conventional exercises as part of their interventions	The analysis within each group reveals a reduction in both ND height and arch index. The average percentage of change was 32.03% for Group A, 35.92% for Group B, and 39.66% for Group C.	The mobilization of the talonavicular joint contributes to the enhancement of joint dysfunction through proprioceptor involvement. Utilizing low dye taping assists in ameliorating foot pronation by anteriorly and medially manipulating the calcaneus, thereby restricting hindfoot eversion along with the linked talar adduction and plantarflexion. The application of a faradic foot bath demonstrated enhancements in the intrinsic muscles of the foot
Kaur et al. (2016) [20]	An observational study (30 individuals with unilateral or bilateral flatfoot)	Staheli’s arch index	Electrical stimulation with the help of a faradic foot bath was given for three weeks	After statistical analysis, it was found that there was a remarkable improvement in Staheli's arch index	The utilization of a faradic foot bath has proven effective in enhancing the MLA among individuals with flat feet
Kim and Kim (2016) [21]	An observational study (14 university students with unilateral or bilateral flatfoot)	NH, Y balance test	The experimental group was given SFE and the control group was given medial arch insoles for five weeks	The analysis of intra-group comparison for the NH and Y balance test revealed a decrease in both values in the experimental group	SFE is found to be effective in decreasing NH and treating flatfoot in individuals
Namsawang et al. (2019) [22]	An RCT (36 participants with flexible flatfoot)	CSA Abdh, NH, Abdh muscle activity	The experimental group was given SFE with NMES, and the control group received SFE with placebo NMES for four weeks	No statistically significant contrast was observed in terms of NH between the experimental and control groups. However, a minor elevation in NH was evident in both groups (0.04 mm for SFE and 0.09 mm for SFE with NMES)	Though there was no remarkable difference in NH, it showed great improvement in Abdh muscle activity, which helped improve flatfoot
Okamura (2019) [23]	An RCT (with 20 participants with flexible flat feet)	NH, FPI	Exercise group: participants underwent an eight-week program of unilateral SFE. Control group: individuals in this group did not receive any form of intervention during the study period	FPI scores improved significantly	SFE is found to be effective in enhancing flat feet along with improving gait kinetic parameters
Ramchandra et al. (2019) [24]	A randomized controlled trial (with 86 pregnant women)	Foot Dysfunction Questionnaire, NH	The study participants were provided with exercises for the feet as well as antenatal exercises. The control group, on the other hand, followed their usual ANC exercises	Intergroup analysis scores were found to be improved between both groups after the intervention	It found that foot exercises were found to be effective in pregnant women in terms of MLA, NH, and pain
Moon and Jung (2021) [25]	A single-blind, randomized, controlled trial study (with 32 subjects with flatfoot)	Dynamic balance, H reflex, static balance	Group A: SMT along with SFE. Group B: SMT	The results in both groups were significantly improved (p<0.01)	After statistical analysis, a significant benefit was observed in the study when the combined intervention of SFE with SMT was incorporated into individuals
Brijwasi and Borkar (2022) [26]	An RCT (52 subjects with flexible flatfoot)	NH, longitudinal arch angle	The participants in the experimental group underwent a six-week period during which they engaged in exercises encompassing foot movements and stretching. In contrast, the control group engaged in active dorsiflexion and plantarflexion exercises exclusively for a duration of six weeks	After six weeks of intervention, it was found that the experimental group showed improvement in NH by 0.4 cm than the control group, and it also showed improvement in longitudinal arch height by 16 degree	It was found that foot exercises given for six weeks were found effective in subjects with flatfoot and also improved the cosmetic appearance of the foot
Hara et al. (2023 ) [27]	Systemic review		Reviewed studies RCT	After reviewing articles, it was found that SFE was useful	After analysis, it was found that SFE is an intervention that is found to be effective
Elsayed et al. (2023 [28]	An RCT (40 participants with symptomatic flexible flatfoot)	ND	The experimental group was given SFE along with shoe insole, and the control group was given shoe insole for six weeks	After intergroup analysis, it was found there was a decrease in NH in the experimental group and there was a decrease in pain	The results show that adding SFE to a shoe insole to treat symptomatic flexible flatfoot reduces foot pain, improves LE function, and changes static and dynamic foot pressure more effectively than using a shoe insole alone
Utsahachant et al. (2023) [29]	An RCT (45 participants with flatfoot)	ND, CPEI	Group A: SFE. Group B: SFLE. Group C: control intervention	The groups that received SFE and SFLE showed significant improvement in NH and greater changes in CPEI	SFE is proven to efficiently improve MLA height and also improve foot propulsion in the stance phase of gait

Discussion

Hara et al. conducted a comprehensive review to assess the effects of SFE on flatfoot deformity. They screened a total of 21 studies, ultimately including nine that met their predetermined criteria, while the remaining 12 were excluded due to inappropriate interventions. The study's outcomes indicated that engaging in SFE indeed results in the contraction of plantar muscles, which, in turn, reduces foot length. This muscle contraction not only pulls the first metatarsal toward the heel but also elevates the MLA, all achieved without flexing the toes. The primary objective of SFE is to fortify the intrinsic foot muscles, which play a crucial role in sustaining the MLA in cases of flatfoot deformity. The study underscored the demanding nature of these exercises, emphasizing the need for patients to have a solid understanding of their execution. Furthermore, the authors noted that the research investigating the effects of SFE on MLA improvement in individuals with flat feet has yielded favorable results [27].

Huang et al. discovered that, despite no significant difference in muscle hypertrophy, their meta-analysis demonstrated the effectiveness of SFE in restoring foot alignment to a more normalized state compared to alternative interventions. Employing a random effects model, they calculated mean differences (MD) and standard mean differences in a meta-analysis that included six trials. From a pool of 609 records, 201 patients met the selection criteria. Within the control group, five out of six trials utilized distinct interventions, such as shoe insoles and muscle-strengthening exercises, while the remaining trial did not specify the intervention. The SFE group exhibited a noticeable reduction in the navicular drop test values (MD: -0.23; 95% p=0.04) and the foot posture index (FPI-6) score (MD: -0.67; 95% p=0.0001) compared to the control group. Consequently, the study suggests that SFE is an effective approach for addressing foot alignment issues in individuals with flat feet and can be seamlessly integrated into rehabilitation or activities that require enhanced foot performance. The research indicated a discernible reduction in foot pronation [3].

In the study conducted by D'silva et al., consistent findings were observed across all groups. When comparing the results within each group, there was a significant and noteworthy decrease in navicular drop height. Specifically, in Group C, there was a statistically significant change in the linear distance of the foot after the treatment. However, the arch index remained unchanged following interventions, including mobilization, low dye taping, and faradic foot bath. When comparing these parameters between the groups, no statistical significance was noted. Following the intervention, Group A exhibited a 32.03% reduction in navicular drop height, Group B showed a 35.92% decrease, and Group C demonstrated a 39.66% reduction. As for the changes in the arch index, Group A remained at 0, Group B at 0.0005, and Group C at 0.002. These results suggest that talonavicular mobilization, faradic foot bath, and low dye taping led to an equally effective reduction in navicular drop height among individuals with flat feet [17].

As per the findings from Kaur et al.'s study, it was demonstrated that the faradic foot bath effectively improved the MLA in individuals with flexible flat feet. The study involved a sample of 30 participants aged between 18 and 28 years. Assessment was performed using Staheli's arch index, and a comprehensive intervention was administered to all participants over a three-week period. Evaluations were conducted at both the beginning and end of the three-week intervention. Importantly, measurements of Staheli's arch index before and after the treatment for subjects with flexible flat feet exhibited a statistically significant difference. The faradic foot bath showed the ability to reduce foot pronation in individuals with flexible flat feet and contribute to the maintenance of the MLA [20].

In a study conducted by Park and Park, the impact of SFE on the muscle activity of the abductor hallucis and navicular height was investigated in individuals both with and without flat feet. The study encompassed 12 subjects, including those with and without pes planus (flat feet). During the SFE, the activity of the tibialis anterior, fibularis longus, and abductor hallucis longus muscles was assessed in both groups, and the navicular drop height was measured before and after the exercises. For the symptomatic group, the navicular drop height was found to be significantly reduced in the post-measurement compared to the pre-measurement. Additionally, during the SFE, the pes planus group exhibited noticeably lower activity in the fibularis longus muscle than the control group (p<0.05). This study concluded that SFE effectively reduced navicular drop in individuals with flat feet [30].

## Conclusions

In conclusion, the review article highlights the significance of assessing the impact of therapeutic interventions such as faradic foot baths and SFE on individuals with symptomatic flat feet. The analyzed studies underscore the potential benefits of these approaches in improving foot arch function and posture, reducing pain, and enhancing overall foot health. However, further well-designed research is warranted to establish the long-term effectiveness, optimal protocols, and comparative efficacy of these interventions. Clinicians and researchers alike should consider the findings of this review as a foundation for shaping future interventions and advancing the management of symptomatic flat feet.
